# Food Hygiene Practices and Associated Factors Among Food Handlers Working at Public Food and Drink Establishments in Mizan‐Aman Town, Bench‐Sheko Zone, Ethiopia, 2023: A Cross‐Sectional Study Design

**DOI:** 10.1002/hsr2.71847

**Published:** 2026-02-22

**Authors:** Abel Girma, Smegnew Gichew, Rahel Dereje, Ermias Ayelew, Buzayehu Alemayehu

**Affiliations:** ^1^ School of Public Health, College of Medicine and Health Science Mizan‐Tepi University Mizan‐Aman Town Ethiopia; ^2^ Department of Midwifery, College of Medicine and Health Science Mizan‐Tepi University Mizan‐Aman Town Ethiopia; ^3^ Departements of Environmental Health, College of Medicine and Health Science Mizan‐Tepi University Mizan‐Aman Town Ethiopia

**Keywords:** food establishments, food handlers, food hygiene, southwest Ethiopia region

## Abstract

**Background and Aims:**

Foodborne illnesses are widespread in both developed and developing countries. Nonetheless, there is a paucity of data about food hygiene practices among food handlers in public catering establishments in Ethiopia, particularly in the study area. Consequently, this research seeks to evaluate the extent of food hygiene practices and the associated factors among food handlers in public food and beverage establishments in Mizan‐Aman town, Southwest Ethiopia, in 2023.

**Methodology:**

This study employed an institution‐based cross‐sectional design involving 372 food handlers. Participants were selected using a simple random sampling technique from individuals employed at randomly chosen public food catering establishments. The data were subsequently entered into Epi Data Version 3.02 and exported to SPSS Version 20 for further analysis. A binary logistic regression analysis was performed. Variables with a *p*‐value of less than 0.25 were included in a multiple binary logistic regression model. Significant factors were ultimately determined based on a 95% confidence interval (CI) and a *p*‐value of less than 0.05.

**Results:**

The prevalence of good food hygiene practices among food handlers working in public food and drink establishments in the study area was 64.6%. An average monthly income of ≥ 1100 ETB (AOR = 5.22; 95% CI = 2.40–11.34, *p*‐value < 0.001), attendance at training (AOR = 4.21; 95% CI = 1.74–10.17, *p*‐value = 0.001), and the availability of a separate dressing room (AOR = 4.89; 95% CI = 1.88, 12.72, *p*‐value = 0.001) were significantly associated with good food hygiene practices.

**Conclusion:**

This study showed the prevalence of good food hygiene practices among food handlers who work in public food and drink establishments in Mizan‐Aman town was low. The variables such as average monthly income of ≥ 1100 ETB, ever attended training on food hygiene, and having a separate dressing room in the facility were significantly associated factors for good food hygiene practice in the study area.

**Abbreviations**
AOR: adjusted odd ratio
COR: crude odd ratio
ETB: Ethiopian Birr
FAO: Food and Agriculture Organization
FBD: food borne disease
SNNPR: Southern Nations, Nationalities, and Peoples' Region
WHO: World Health Organization

## 1 Introduction

Food hygiene involves all the conditions and measures necessary to ensure the safety and appropriateness of food at every stage of the food chain [1]. As a fundamental human need, food is essential for maintaining good health, providing vital energy, vitamins, and minerals. Failing to maintain proper hygiene standards in food storage and preparation can negatively impact human health. This issue can stem from various factors, including inadequate knowledge, poor handling practices, and negative attitudes among food handlers in food and beverage establishments [2].

Worldwide, the rapid growth of urbanization, industrialization, the tourism sector, and economic globalization has contributed to a significant increase in the number of food establishments [3, 4, 5, 6]. This trend is evident in Ethiopia, particularly in Mizan‐Aman, one of the capital towns in the southwest region of Ethiopia, where the number of food establishments is increasing at an accelerated pace. This growth may pose challenges to food hygiene, as hygiene regulators might struggle to keep pace with the rapid expansion and may, in some instances, lack the necessary authority to enforce food hygiene standards. Consequently, food establishments may experience a rise in the incidence of foodborne illnesses.

Foodborne diseases (FBD) are common in both developed and developing countries [7, 8, 9, 10, 11, 12]. Approximately 70% of diarrheal disease cases in developing nations are linked to the consumption of contaminated food [13]. Annually, around 600 million people fall ill due to contaminated food, leading to an estimated 420,000 deaths [14]. The World Health Organization (WHO) reports that one in 10 individuals worldwide suffers from foodborne illnesses [15]. In 2010, foodborne infectious diseases were estimated to affect 550 million people and cause approximately 230,000 deaths globally; however, determining the precise mortality rate attributable to FBD remains challenging [16].

The predominant risk factors contributing to foodborne illness include inadequate personal hygiene, improper food handling practices, and contaminated food surfaces and equipment [5, 17]. Food handling practices are a primary source of food contamination. Various evidence suggests that inadequate food handling by food handlers accounts for 10%–20% of FBD [8, 18, 19].

In developing countries, particularly in Ethiopia, food hygiene practices are notably poor, as indicated by previous studies. For example, only half (50.2%) of food handlers in Ethiopia demonstrated adequate food hygiene practices, with significant regional variations observed among food handlers [20]. The Southern Nations, Nationalities, and Peoples' Region (SNNPR) recorded the lowest level of good food hygiene practices, at just 32.6% [20].

Prior research has recognized a range of factors that influence the adherence to effective food hygiene practices among food handlers in developing nations. Several determinants, encompassing individual, institutional, and socioeconomic variables, impact the capacity of food handlers to prepare and manage food in a hygienic manner [6, 21, 22]. In Ethiopia, specific predictors of good food hygiene practices have been identified, such as age, presence of supervisors, marital status, monthly income, work experience, routine medical checkups, training, knowledge, gender, education, attitude, availability of shower facilities, separate dressing rooms, and hand washing facilities [20, 23, 24, 25, 26, 27, 28, 29].

Despite significant efforts to address public health issues in Ethiopia food hygiene practices among food handlers continue to pose ongoing challenges [30]. There is limited data available on the prevalence of FBD linked to inadequate food safety in food and drink service establishments in Ethiopia [24]. Mizan‐Aman, the capital of the Bench‐Sheko Zone administration, is rapidly urbanizing, leading to increased reliance on food, beverage, and accommodation services by the population. Therefore, evaluating and disseminating information about the food handling practices of workers and the factors influencing them is crucial for enhancing food hygiene practices among handlers within food and drink service establishments in this region. Additionally, to the best of the researcher's knowledge, there have been no prior studies examining the impact of food handlers on public food catering services in this context. Therefore, this study aimed to answer the question, what is the level of food hygiene practice and associated factors among food handlers working at public food and drink service establishment in Mizan‐Aman town, Southwest Ethiopia, 2023.

## 2 Methods

### 2.1 Study Area and Period

The research was undertaken in Mizan‐Aman, the administrative center of the Bench‐Sheko Zone, which is situated 562 km from Addis Ababa. According to the Zonal annual reports of 2019, Mizan‐Aman has a total population of 58,364 individuals and comprises nine kebeles. Within the newly formed Bench‐Sheko Zone, there are 28 public health institutions, including 26 public health centers, one primary hospital, and one general hospital [31]. Additionally, it was estimated that there are 342 licensed and regulated food and beverage establishments in Mizan‐Aman, employing a total of 2452 food handlers [32]. The study was conducted between April and May 30, 2023.

### 2.2 Study Design

An institution‐based cross‐sectional study design was employed.

### 2.3 Source Population

All food handlers who worked at licensed public food and drink establishments (Hotels, restaurants, butcher houses, grocery, cafeterias, cafeterias and restaurants, breakfast houses) in Mizan‐Aman Town, Bench‐Sheko Zone, Southwest Ethiopia.

### 2.4 Study Population

All food handlers working at the selected public food and drink establishments in Mizan‐Aman Town.

### 2.5 Study Unit

All selected individuals who work in selected establishments.

### 2.6 Inclusion Criteria

All food handlers who were working at public food catering establishments.

### 2.7 Exclusion Criteria

Too sick or mentally unstable to respond to questions.

### 2.8 Sample Size Determination

In this study, the sample size was determined by using a single population proportion formula. By considering the prevalence of good food hygiene practice is 32.6% [23], 5% margin of error, a 95% confidence level, and a non‐response rate of 10%. Based on this, the actual sample size was: *n *= (*zα*/2)2 *p* (1–p)/*d*2 [33]= (1.96)^2^*0.326(1–0.326)/0.05^2^, *n* = 338. Thus, a minimum number of 338 food handlers were required in the study. Then, a 10% non‐response rate was considered, and the minimum sample size required became: 338+ (338*10%) = 372. Hence, the final sample size of this study was 372.

### 2.9 Sampling Technique and Procedure

Out of a total of 342 public food and drink establishments, which include Hotels, restaurants, cafeterias, butcher houses and grocery, breakfast houses, and cafeterias and restaurants in Mizan‐Aman town, about 105 establishments were selected by using simple random sampling techniques. The calculated sample size was allocated proportionally to each selected establishment based on the total number of food handlers they contain and finally, the study participants were selected by lottery methods from each establishment.

### 2.10 Study Variables

The dependent variable of this study was food hygiene practice and the independent variables were socio‐demographic variables (age, sex, monthly income, educational status, marital status, work experience, and job types), institutional and related factors (type of the food establishment, availability of hand washing facility, toilet facilities, dressing rooms, shower facilities Protective personal equipment's, and presence of supervisor), training on food safety, knowledge on food safety, attitude toward food hygiene practice, and inspection from health professionals.

### 2.11 Data Collection Procedures

A structured, interviewer‐administered questionnaire and observational checklist were employed as the instruments for data collection. The validated questionnaire was adapted from previously published researches [24, 28, 34]. It encompassed inquiries regarding socio‐demographic characteristics, institutional factors, training related to food safety, knowledge of food hygiene, attitudes toward food hygiene practices, and actual food hygiene practices.

The questionnaire was organized into several distinct sections. The first section aimed to gather information about the socio‐demographic characteristics of food handlers, while the second section focused on institutional and related factors affecting food handlers. The third section addressed the knowledge status of food handlers regarding hygienic practices, drawing upon insights from earlier studies [24, 28, 34]. This section specifically examined respondents' awareness of personal hygiene, food contamination, FBD, temperature control, and the modes of transmission associated with foodborne illnesses.

A total of 24 questions were employed to evaluate knowledge regarding food safety, with responses coded as 0 for “No” and 1 for “Yes.” The scoring system for food hygiene knowledge ranges from 0 to 24 [34]. Section four comprised attitude questions related to food hygiene, which were assessed through a total of 22 questions items utilizing a five‐point Likert‐type scale (coded as 1 for “strongly disagree,” 2 for “disagree,” 3 for “neutral,” 4 for “agree,” and 5 for “strongly agree”) to gauge the attitudes of food handlers toward food hygiene. The attitude scores span from 22 to 110 [34].

In section five, which focuses on food hygiene practices, the respondents' adherence to good hygienic practices was assessed through an observational checklist addressing personal hygiene and other safe food handling behaviors [24, 28, 34]. This section consisted of 23 questions/statements or checklist items, each offering two possible responses: “Yes” or “No,” which were coded as 0 for incorrect practices and 1 for correct practices. The scoring for food hygiene practices ranges from 0 to 23 [34].

### 2.12 Data Quality Assurance

The data collection process was conducted by three proficient BSc nurses with expertise in field data collection, alongside one specialist in food safety and environmental sanitation inspection. To ensure the quality of the collected data, several measures were implemented, including pre‐testing, translation of the data collection instrument into the local language (Amharic), and subsequent back‐translation to English for consistency verification. Additionally, comprehensive training was provided to the data collectors and their supervisors. Furthermore, a day‐to‐day follow‐up was undertaken during data collection by the field supervisor and researchers. Subsequently, the gathered data were assessed for completeness and underwent cleaning before analysis.

### 2.13 Data Analysis Methods

Data were coded and entered into Epi Data Version 3.02 and exported to a Statistical Package for Social Science (SPSS) version 20 for analysis. The descriptive statistics such as frequencies, percentage, mean, and standard deviation (SD) were calculated for variables of interest, and the results were presented using texts and tables. Bivariate logistic regression analysis was conducted to examine the association between dependent and independent variables. All variables with *p* < 0.25 in bivariate analysis were entered into the multiple logistic regression model to control all possible confounders and identify factors independently associated with food hygiene practice [35]. To measure the strength and direction of association between independent and dependent variable, crude odd ratio (COR), and adjusted odd ratio (AOR) with a 95% confidence interval (CI) were used. Finally, the variable that shows statistical significance (*p*‐value < 0.05 cut point) in multivariate analysis was considered statistically significant. The multicollinearity between independent variables was checked by using the standard error (SE), and variables of SE > 2 were dropped from the analysis. The model fitness was tested by Hosmer– Lemeshow for the goodness of fit, and the model fit was considered at Hosmer–Lemeshow *p*‐value > 0.05 [35, 36].

### 2.14 Operational Definition

**Food hygiene practice:** Poor food hygiene practice is defined as a score of less than 60% on food hygiene practice‐related questions [34]. Conversely, good food hygiene practice is identified when food handlers score 60% or higher on these questions and are categorized as having a “good level of food hygiene practice” [34].

**Knowledge:** a score of less than 60% on food hygiene knowledge‐related questions, or fewer than 15 correct answers out of 24 total questions, signifies poor knowledge of food hygiene [34]. In contrast, those who scored 60% or more were classified as having a “good level of knowledge regarding food hygiene” [34].

**Attitude:** Unfavorable attitudes are characterized by respondents who score below 60% on the attitude‐related questions, while those who score 60% and above on the attitude score were considered as having a “favorable attitude towards food hygiene [34].

**A food handler:** defined as anyone working in a food and beverage establishment who interacts with food or comes into contact with equipment or utensils that are likely to touch food [37].

**Food and drink establishments:** are institutions that serve meals and beverages to a large number of customers, offering options like breakfast, lunch, and dinner, as well as drinks [38].

### 2.15 Ethical Approval

This study was conducted according to the Declaration of Helsinki. After approval of the proposal, a formal institutional collaboration letter was obtained from the Mizan‐Tepi University Research Directorate office and submitted to the respected study institutions. After a brief description of the study objectives, purpose, confidentiality, and the benefits of the study permission was sought from the study institution to start the data collection. During the data collection period, it was also explained to the participants that there was no intention to collect additional information from the study participants outside of the stated objectives. It was also explained that the results will be kept confidential.

## 3 Results

### 3.1 Socio‐Demographic and Economic Characteristics of the Study Participants

A total of 372 food handlers, who worked in food and drink establishments, were included in this study, with a response rate of 95%. The mean age of the respondents was 23.6 (±4.7), while the majority (79.7%) were in the age range of 18–29 years old. Around 25.8% of the respondents earned an average monthly income of less than 1100 ETB. Only around half (51%) of the participants had work experience of greater than and above 2 years in the establishments (Table 1).

**Table 1:** Socio‐demographic and economic characteristics of study participants working at a public food and drink establishment in Mizan‐Aman town, Bench‐Sheko zone, Ethiopia, 2023 (*N* = 353).

Variables	Frequency	Perce cent (%)
Age		
< 18 years	43	12.2
18–29 years	282	79.9
≥ 29 years	28	7.9
Sex		
Female	265	75.1
Male	88	24.9
Marital status		
Married	106	30
Single	238	67.4
Divorced	9	2.5
Educational status		
Can't read and write	18	5.1
Can read and write	49	13.9
Primary	105	29.7
Secondary	100	28.3
Tertiary	81	22.9
Average monthly income		
< 1100 ETB	91	25.8
≥ 1100 ETB	262	74.2
Work experience		
< 2 years	173	49.0
≥ 2 years	180	51.0
Types of job		
Cooker	144	40.8
Waiter	188	53.3
Both	21	5.9

### 3.2 Institutional and Related Factors of Food Handlers

A total of 105 food and drink establishments were visited. Regarding the facility‐related factors, among the 353 food handlers, around 55 (15.6%) and 85 (24.4%) of food handlers had been working in hotels and breakfast houses, respectively. About 209 (59.2%) of food handlers had not ever attended training on food safety, while 22.4% of the facility had no personal protective equipment. The majority, 77.4% of the establishments had not received supervision from the regulatory personnel (Table 2).

**Table 2:** Institutional and related characteristics of study participants working in public food and drink establishments in Mizan‐Aman town, Bench‐Sheko zone, Ethiopia, 2023 (*N* = 353).

	Number of respondents	Percent (%)
Type of establishment		
Hotel	55	15.6
Restaurant	67	19
Cafeteria and Restaurant	35	9.9
Bucher house and grocery	48	13.6
Breakfast house	63	17.8
	85	24.1
Ever attended food safety training		
Yes	144	40.8
No	209	59.2
Attended food safety training in the last 2 years		
Yes	86	24.4
No	267	75.6
Presence of hand washing facility in the food establishment		
Yes	334	94.6
No	19	5.4
Availability of soap with the hand washing facility		
Yes	337	95.5
No	16	4.5
Availability of personal protective equipment (hair cover, mask, alcohol, and glove)		
Yes	274	77.6%
No	79	22.4%
The presence of pipe water in the kitchen area		
Yes	230	65.2
No	123	34.8
The presence of a supervisor in the facility		
Yes	157	44.5
No	196	55.5
Separate dressing room for food handlers		
Yes	288	81.6
No	65	18.4
Get supervision from the regulatory personnel (health professionals)		
Yes	79	22.4
No	274	77.6
Availability of toilet		
Yes	345	97.7%
No	8	2.3%
Types of toilet		
Water carriage	152	43.1
Dry latrine	201	56.9

### 3.3 Knowledge of Food Handlers Regarding Food Hygiene and Safety

The magnitude of good knowledge on food hygiene among food handlers working in food and drinks establishments in Mizan‐Aman town was 94.3% (95% CI = 91.8, 96.6%).

### 3.4 The Attitude of Food Handlers Regarding Food Hygiene and Safety

Among a total of 353 food handlers working in food and drinks establishments in Mizan‐Aman town, all of them (100%) had a good attitude toward food hygiene practices.

### 3.5 Food Hygiene Practice

Among a total of 353 food handlers working in public food and drink establishments in Mizan‐Aman town, 64.6% (95% CI = 59.5%, 69.4%) had good food hygiene practices.

Factors associated with food hygiene practice. In binary logistic regression analysis, the variables such as educational status, average monthly income, the types of jobs, ever‐attended training, presence of pipe water in the kitchen, presence of a supervisor, and availability of a separate dressing room were significantly associated with the outcome variable. However, in multiple binary logistic regression analyses after adjustment for possible confounder's, three variables such as average monthly income, ever‐attended training on food hygiene, and availability of separate dressing rooms, were significantly associated with food hygiene practice among food handlers working in food and drink establishments (Table 3).

**Table 3:** The binary and multiple binary logistic regression analysis to identify factors associated with food hygiene practice among food handlers working at food and drink establishments in Mizan‐Aman town, Bench‐sheko zone, Ethiopia, 2023 (*N* = 353).

Variables	Food hygiene practice		COR (95% CI)	*p* value	AOR (95% CI)	*p* value
	Number of poor (%)	Number of goods (%)				
Educational status	35 (9.9)					
No formal education	49 (13.9%)	32 (9.1%)	1		1	
Primary	41 (11.6%)	56 (15.9%)	1.25 (0.67–2.31)	*p* = 0.48	0.34 (0.105–1.099)	*p* = 0.07
Secondary and above		140 (39.7%)	3.73 (2.06–6.75)	*p* < 0.001	0.996 (0.39–2.54)	*p* > 0.99
Average income level						
< 1100 ETB	59 (16.7%)	32 (9.1%)	1		**1**	
≥ 1100 ETB	66 (18.7%)	196 (55.5%)	5.47 (3.28–9.14)	*p* < 0.001	5.22 (2.40–11.34)	*p* < 0.001
Types of job						
Cookers	67 (19%)	77 (21.8%)	1		1	
Waiters	49 (13.9%)	139 (39.4%)	2.43 (1.53–3.86)	*p* < 0.001	0.92 (0.34–2.53)	*p* = 0.87
Both cooker and waiters	9 (2.5%)	12 (3.4%)	1.14 (0.45–2.881)	*p* = 0.78	0.45 (0.09–2.21)	*p* = 0.33
Ever got training on food hygiene						
Yes	29 (8.2%)	115 (32.6%)	3.37 (2.06–5.498)	*p* < 0.001	4.21 (1.74–10.17)	
No	96 (27.2%)	113 (32%)	1		1	*p* = 0.001
The presence of pipe water in the kitchen area						
Yes	62 (17.6%)	168 (47.6%)	2.85 (1.80–4.498)	*p* < 0.001	1.09 (0.39–3.00)	*p* = 0.87
No	63 (17.8%)	60 (17%)	1		1	
The presence of a supervisor in the facility						
Yes	34 (9.6%)	123 (34.8%)	3.14 (1.95–5.03)	*p* < 0.001	1.16 (0.60–2.31)	*p* = 0.66
No	91 (25.8%)	105 (29.7%)	1			
Availability of separate dressing						
Yes	82 (23.2%)	206 (58.4%)	4.90 (2.77–8.72)	*p* < 0.001	4.89 (1.88–12.72)	*p* = 0.001
No	43 (12.2%)	22 (6.2%)	1		1	

Abbreviations: AOR, adjusted odd ratio; COR, crude odd ratio; ETB, Ethiopian Birr.

The odds of good hygiene practice were approximately five times higher among food handlers who earned greater or equal to 1100 ETB average monthly income as compared with the odds among the food handlers who earned less than 1100 ETB of average monthly income (AOR = 5.22; 95% CI = 2.40–11.34, *p* value < 0.001). The odds of good food hygiene practice were approximately 4.21 times higher among food handlers who were working in food and drink establishments that ever attended training on food hygiene or safety as compared with the odds of food hygiene practice among the food handlers who are working in the public food and drink establishment that does not ever attended training on food hygiene (AOR = 4.21; 95% CI = 1.74, 10.17, *p* value = 0.001).

The odds of good food hygiene practice among food handlers who had a separate dressing room were approximately 4.89 times higher good than their counterparts (AOR = 4.89; 95% CI = 1.88, 12.72, *p* value = 0.001) (Table 3).

## 4 Discussion

This study found a prevalence rate of good food hygiene practices among food handlers in public food and drink establishments of 64.6%, which aligns with results from similar research conducted in various regions of Ethiopia, including Asosa (67.8%) [39], Bahir Dar (67.5%) [40], and Gondar (66.6%) [41]. However, it was higher than the study findings conducted at Debre Markos town (54%) [42], East Gojjam and West Gojjam Zones (48.8%) [34], in Arba Mich town (32.6%) [23], 27.4% in Bole sub‐city, Addis Abeba [43] and in Woldia (46.5%) [24] and Dangila towns (52.5%) [28] of food handlers had a good food handling practice. This variation could be due to the measurement tools and cut‐off points used to determine the food hygiene practice of the study participants (60% vs. 80%). It may also be due to study period variation, while some of the previous studies were conducted during the COVID‐19 pandemic, during which some rules and regulations of food hygiene practice were enforced. In addition, the variation might be due to environmental and socioeconomic factors.

The findings of this study showed that food handlers who earned an average monthly income greater or equal to 1100 ETB were associated with good food hygiene practice. This is in line with the study conducted in Dangila town [28], Gonder town [44], and Woldia town, Northwest Ethiopia [24]. The possible reason for this might be that those who had a monthly income ≥ 1100 ETB might have good educational status, experience, and knowledge of food handling practices. Moreover, those individual who earn higher might be keep their personal hygiene and they could be afford sanitary commodities which increase their food handling practice.

According to this study finding, ever attending training on food hygiene or safety were significantly associated with the good food hygiene practice. It is consistent with the study conducted in Arba‐Minch Town, Gammo Gofa Zone [23], and in Debre‐Markos town [42]. This is due to the fact that when food handlers are properly trained, they can take the necessary precautions to avoid malpractice in food handling. In addition to that getting training about food handling practice might be enhance the knowledge and attitude of food handlers and increase good food hygiene practice. Therefore, food handlers should attend proper training in the basic principles of food safety and rules of personal hygiene in order to improve their practices in food handling.

This study revealed that food handlers who had separate dressing rooms were positively associated with good food hygiene practice. This finding aligns with previous studies conducted in Dangila town [28] and in East Gojjam and West Gojjam Zones [34]. A plausible explanation for this observation is that food handlers in establishments with dedicated dressing rooms are likely to maintain a cleaner working environment, thereby promoting better food handling practices.

### 4.1 Limitations of the Study

Social desirability bias and not including microbial assessment could be the main limitations of this study. In addition, due to the nature of the study design, this study was unable to show a cause–effect relationship.

## 5 Conclusion

This study indicates that the prevalence of proper food hygiene practices among food handlers was insufficiently low when compared with the previous studies. Factors such as an average monthly income of ≥ 1100 ETB, prior training in food hygiene, and the availability of a designated dressing room within the facility were found to be significantly associated with positive food hygiene practices in the region. Consequently, it is imperative to enhance the adoption of good food hygiene practices among food handlers in Mizan‐Aman town. The health departments within the Bench‐Sheko Zone should implement ongoing food safety training for all food handlers. Additionally, the Mizan‐Aman town municipal bureau should mandate that each establishment provide a separate dressing room and training about food hygiene handling practice for food handlers. Furthermore, we recommend that future research include microbial assessments to gain a more comprehensive understanding of food safety practices in this context. In addition to that to compare and contrast the result, the future researcher should also use a standardized cut‐off put.

## Author Contributions

**Abel Girma:** conceptualization, investigation, funding acquisition, writing – original draft, methodology, writing – review and editing, formal analysis, supervision. **Smegnew Gichew:** writing, reviewing, editing, methodology, validating. **Rahel Dereje:** investigation, funding acquisition, methodology, data curation **Ermias Ayelew:** funding acquisition, validation, writing — review and editing, project administration. **Buzayehu Alemayehu:** investigation, methodology, resources, supervision, data curation, funding acquisition.

## Consent

Written informed consent was sought from each study participant before starting data collection according to the national legislation and institutional guideline.

## Conflicts of Interest

The authors declare no conflicts of interest.

## Transparency Statement

The lead author Abel Girma affirms that this manuscript is an honest, accurate, and transparent account of the study being reported; that no important aspects of the study have been omitted; and that any discrepancies from the study as planned (and, if relevant, registered) have been explained.

## Data Availability

The data and materials are available in the corresponding authors institution and will be made available upon formal request. The corresponding author (Abel Girma) had full access to all of the data in this study and take complete responsibility for the integrity of the data and the accuracy of the data analysis.
